# State Feedback Optimal *L*_2_-Induced Control of Nonlinear Systems Utilizing Universal Approximation

**DOI:** 10.3390/e28050531

**Published:** 2026-05-07

**Authors:** Adrian-Mihail Stoica, Isaac Yaesh

**Affiliations:** 1 Faculty of Aerospace Engineering, National University of Science and Technology POLITEHNICA of Bucharest, 011061 Bucharest, Romania; 2 Control Department, Elbit Systems, Ramat-Hasharon 47100, Israel; isaac.yaes@elbitsystems.com

**Keywords:** sector-bounded nonlinearities, *L*_2_-induced norm boundedness conditions, state feedback control, conservatism reduction, universal approximation theorem, van der Pol forced oscillator

## Abstract

This paper presents an optimal $L_{2}$-induced control problem for systems with multiple sector-bounded nonlinearities. Sufficient boundedness conditions for the $L_{2}$-induced norm are derived in terms of a specific system of linear matrix inequalities (LMIs). Based on these conditions, an optimal state feedback control problem is then formulated and solved for the considered class of nonlinear systems. A procedure to reduce the conservatism of the derived conditions is also provided. The proposed formulation, which explicitly considers multiple sector-bounded nonlinearities, is useful because it enables optimal $L_{2}$-control problems for a much wider class of nonlinearities. Indeed, by invoking the universal approximation theorem, one may represent nonlinearities that do not satisfy sector-bounded conditions as a weighted sum of sector-bounded sigmoid functions. The theoretical and procedural developments are illustrated by a numerical example consisting of the state feedback optimal $L_{2}$-induced control of a forced van der Pol oscillator.

## 1. Introduction

The control of nonlinear plants has been a challenging problem to the control community for many years. A plethora of methods and approaches have been developed over the years, and here we cite just a few. An approach that generalizes linear systems is presented in [1], where a nonlinear plant is considered, but within the class of Lipschitz nonlinear systems. The method utilizes the Lipschitz bounds to derive LMIs that guarantee stability and $H_{\infty }$ performance. Another approach that is confined to another specific class relates to Lur’e [2]-type sector-bounded [3] nonlinear systems, whereas [4] deals with stochastic, in probability, stabilization of the SAR (Stochastic Anti Resonance, see also [5,6]), whereas [7] deals with the deterministic case. It should be noted that sector-bounded systems strongly relate to Hopfield networks [8]. The present paper focuses on the deterministic continuous-time problem and is aimed at bounded-real-lemma-like characterization of the $L_{2}$-induced norm (actually $H_{\infty }$-norm in linear systems) and its application for control synthesis. A related discrete-time problem has been considered in [9].

We note that the scope of applications of Lur’e-type systems control is much wider than what may seem to be at first sight. This wider scope is enabled by the universal approximation theorem [10] for systems that are not a priori modeled with sector-bounded uncertainties. More precisely, when the model is not a priori sector-bounded, one may invoke the universal approximation theorem to fit a neural network with a single hidden layer, with, e.g., a tanh activation function and a linear output layer. Such networks provide an approximation with arbitrarily small error for a wide enough hidden layer. Consequently, the model of the system is readily and closely approximated as a sector-bounded Lur’e system. In the present paper it is shown that using the state feedback optimal control approach developed for the sector-type nonlinearities in combination with Cybenko’s universal approximation theorem, one can derive $L_{2}$-induced norm boundedness conditions for dynamic systems with wider classes of nonlinearities. This procedure is illustrated in Section 6 for the optimal $L_{2}$-induced norm control of a van der Pol oscillator whose nonlinear term is not of bounded-sector type. The model of this system has been intensively studied in the context of the control of nonlinear oscillators. For instance, in [11], global stabilization of the van der Pol system is analyzed. Different approaches to control such systems have been proposed, including PID (Proportional-Integral-Derivative) controllers (see e.g., [12]), optimal controllers (e.g., [13,14]), nonlinear adaptive ([15] and predictive controllers [16]), neural network-based control [17], to mention just a few references from the vast literature devoted to this topic.

The main contributions of this paper are the following:

- Characterization of the $L_{2}$-induced norm boundedness conditions for a class of systems with multiple sector-type nonlinearities;
- Sufficient conditions for the existence of a state feedback controller providing an imposed level of the $L_{2}$ induced norm for systems with sector-bounded nonlinearities;
- A loop transformation procedure to reduce the conservatism of the $L_{2}$ boundedness conditions;
- Use of the universal approximation theorem to approximate general nonlinearities with sums of sector-bounded nonlinearities;
- Illustration of the derived theoretical results for the optimal $L_{2}$ induced norm control of the van der Pol oscillator.

The remainder of this paper is organized as follows: Section 2 presents the control problem formulation, whereas in Section 3 an $L_{2}$-induced norm characterization for systems with multiple sector-bounded nonlinearities is provided. In Section 4, the state feedback optimal $L_{2}$-induced control problem is solved. In order to reduce the conservatism of the boundedness conditions, a loop transformation procedure is proposed in Section 5. Further, using the universal approximation theorem, an $L_{2}$-induced norm control problem is considered for the forced van der Pol oscillator. The paper ends with some final conclusions.

Notation. Throughout the paper, the superscript ‘⊤’ stands for matrix transposition, R denotes the set of scalar real numbers whereas $Z_{+}$ stands for the non-negative integers. Moreover, $R^{n}$ denotes the *n* dimensional Euclidean space, $R^{n\times m}$ is the set of all n×m real matrices, and the notation P > 0 (P≥0), for $P\in R^{n\times n}$ means that *P* is symmetric and positive definite (positive semi-definite). By *I* one denotes the identity matrix of appropriate dimensions. The trace of a matrix *Z* is denoted by Tr(Z), and |v| represents the Euclidian norm of an *n*-dimensional vector *v*. By $L^{2}\left(\left[0,\infty \right),R^{m}\right)$ one denoted the Lebesgue space of all $R^{m}$ valued functions f(t), t∈[0,∞), with the property that $\int_{0}^{\infty }{\left|f\left(t\right)\right|}^{2}dt<\infty$. Finally, note that the terms Lyapunov and Riccati equations in this paper refer to generalized versions of the standard equations appearing in the the $H_{2}$ and $H_{\infty }$ control literature. Further, we denote by vec the operation that stacks all columns of a matrix into $R^{n^{2}}$ and by vech the operator that stacks the lower triangular part of a symmetric matrix into $R^{n(n+1)/2}$. We denote the inverse operation by $vech^{-1}$. Also $\mathrm{diag}(x_{1},\ldots ,x_{n})$ denotes the diagonal matrix with the scalars $x_{i},\;i=1,\ldots ,n$ on its main diagonal.

## 2. Optimal Control Problem Formulation

Consider the following deterministic time-invariant nonlinear system:

(1) $$\begin{array}{c} \begin{array}{ccc} \dot{x}\left(t\right) & = & Ax\left(t\right)+Ff\left(y\left(t\right)\right)+B_{1}w\left(t\right)+B_{2}u\left(t\right)\\ z(t) & = & Mx(t)+Nu(t) \end{array} \end{array}$$

in which t≥0 denotes the time variable and where the vector-valued functions x(t),w(t),u(t),z(t) and y(t) are defined as follows: $x\left(t\right)\in R^{n}$ represents the state variable of the system (1), $w\left(t\right)\in R^{m_{1}}$ is an exogenous input, $u\left(t\right)\in R^{m_{2}}$ denotes the control input, $z\left(t\right)\in R^{p}$ is the system quality output, and $y\left(t\right)\in R^{q}$ stands for the input to the activation function f(·). It is assumed that the nonlinear term $f\left(y\left(t\right)\right)\in R^{q}$ has the following form:

(2) $$\begin{array}{c} f\left(y\right)=\left[\begin{array}{c} f_{1}\left(y_{1}\right)\\ \vdots \\ f_{q}\left(y_{q}\right) \end{array}\right] \end{array}$$

in which $f_{i}:R\to R$ are sector-type nonlinearities satisfying the conditions $f_{i}\left(y_{i}\right)\left(f_{i}\left(y_{i}\right)-\sigma_{i}y_{i}\right)\leq 0$ and where $y_{i}=C_{i}x$ with $C_{i}\in R^{1\times n}$, i=1,…,q. The slope bounds $\sigma_{i}>0,\;$i=1,…,q of the nonlinearities are assumed given and the matrix coefficients $A\in R^{n\times n},\;B_{1}\in R^{n\times m_{1}},\;B_{2}\in R^{n\times m_{2}},\;F\in R^{n\times q},\;C_{i}\in R^{1\times n},\;i=1,\ldots ,q,\;M\in R^{p\times n}$ and $N\in R^{p\times m_{2}}$ are given and time-invariant. Throughout the paper it is assumed that $M^{⊤}N=0$.

The problem consists of determining a state feedback control law u(t)=Kx(t) such that for a given γ>0, the resulting system is stable and $\int_{0}^{\infty }\left(z^{⊤}\left(t\right)z\left(t\right)-\gamma^{2}w^{⊤}\left(t\right)w\left(t\right)\right)dt<0$ for all $w\left(t\right)\in L^{2}\left(\left[0,\infty \right),R^{m_{1}}\right)$. To this end, we first characterize the $L_{2}$-induced norm.

## 3. *L*_2_-Induced Norm Characterization

The following result provides sufficient conditions for the boundedness of the $L_{2}$-induced norm of the system with multiple nonlinearities.

**Lemma** **1.** *Consider the system*

(3) $$\begin{array}{c} \begin{array}{ccc} \dot{x}\left(t\right) & = & Ax(t)+Ff(y(t))+Dw(t)\\ z(t) & = & Lx(t) \end{array} \end{array}$$

*in which the matrix coefficients $A\in R^{n\times n},\;F\in R^{n\times q},\;D\in R^{n\times m_{1}}$ and $L\in R^{p\times n}$ are given and time-invariant. It is assumed that the nonlinearities f(y) have the form (2). If, for a given level of attenuation γ>0 of the induced $L_{2}$-norm, there exist matrices P>0, $\Lambda =\mathrm{diag}(\lambda_{1},\ldots ,\lambda_{q})>0$ and $T=\mathrm{diag}(\tau_{1},\ldots ,\tau_{q})\geq 0$ such that*

(4) $$\begin{array}{c} \left[\begin{array}{ccc} A^{⊤}P+PA+L^{⊤}L & A^{⊤}C^{⊤}\Lambda +PF+\frac{1}{2}C^{⊤}ST & PD\\ {\left(1,\;2\right)}^{T} & \Lambda CF+F^{⊤}C^{⊤}\Lambda -T & \Lambda CD\\ {\left(1,\;3\right)}^{T} & {\left(2,\;3\right)}^{T} & -\gamma^{2}I \end{array}\right]<0 \end{array}$$

*in which $C\in R^{q\times n}$ has the rows $C_{i},\;i=1,\ldots ,q$ and $S:=\mathrm{diag}(\sigma_{1},\ldots ,\sigma_{q})$, then the system (3) is stable and $\int_{0}^{\infty }\left(z^{⊤}\left(t\right)z\left(t\right)-\gamma^{2}w^{⊤}\left(t\right)w\left(t\right)\right)dt<0$ for all $w\left(t\right)\in L^{2}\left(\left[0,\infty \right),R^{m_{1}}\right)$.*

**Proof.**  Consider the Lyapunov function candidate

$$\begin{array}{c} V\left(x\right)=x^{⊤}Px+2\sum_{i=1}^{q}\lambda_{i}\int_{0}^{y_{i}}f_{i}\left(s\right)ds. \end{array}$$

Denoting $\Lambda =\mathrm{diag}\{\lambda_{1},\ldots ,\lambda_{q}\}$, direct computations give

(5) $$\begin{array}{c} \begin{array}{c} \dot{V}\left(x\right)=\left(x^{⊤}A^{⊤}+f^{⊤}F^{⊤}+w^{⊤}D^{⊤}\right)\left(Px+C^{⊤}\Lambda f\right)\\ +\left(x^{⊤}P+F^{⊤}\Lambda C\right)\left(Ax+Ff+Dw\right). \end{array} \end{array}$$

Therefore, $\dot{V}\left(x\right)$ may be expressed as

(6) $$\begin{array}{c} \dot{V}\left(x\right)=\xi^{⊤}F_{0}\xi \end{array}$$

where by notation $\xi :={\left[x^{⊤}\;f^{⊤}\;w^{⊤}\right]}^{⊤}$ and

(7) $$\begin{array}{c} F_{0}:=\left[\begin{array}{ccc} A^{⊤}P+PA & A^{⊤}C^{⊤}\Lambda +PF & PD\\ {\left(1,\;2\right)}^{⊤} & \Lambda CF+F^{⊤}C^{⊤}\Lambda & \Lambda CD\\ {\left(1,\;3\right)}^{⊤} & {\left(2,\;3\right)}^{⊤} & 0 \end{array}\right]. \end{array}$$

Using the norm condition $\int_{0}^{\infty }\left(z^{⊤}\left(t\right)z\left(t\right)-\gamma^{2}w^{⊤}\left(t\right)w\left(t\right)\right)dt<0$ it follows that $dV/dt+z^{⊤}z-\gamma^{2}w^{⊤}w<0$, where the latter condition can be expressed as $\xi^{⊤}\left(F_{0}+F_{w}\right)\xi \leq 0$, with

(8) $$\begin{array}{c} F_{w}:=\left[\begin{array}{ccc} L^{⊤}L & 0 & 0\\ 0 & 0 & 0\\ 0 & 0 & -\gamma^{2}I \end{array}\right]. \end{array}$$

Define

$$\begin{array}{c} F_{c}:=\left[\begin{array}{ccc} 0 & \frac{1}{2}C^{⊤}ST & 0\\ \frac{1}{2}TSC & -T & 0\\ 0 & 0 & 0 \end{array}\right] \end{array}$$

with the diagonal matrix *T* as introduced in the statement. Noticing that $\xi^{⊤}F_{c}\xi =-\Sigma_{i=1}^{q}\tau_{i}f_{i}\left(y_{i}\right)\left(f_{i}\left(y_{i}\right)-\sigma_{i}y_{i}\right)\geq 0,$ in accordance with the S procedure for quadratic terms and strict inequalities (see e.g., [3]), it follows that, if there exists $\tau_{1}\geq 0,\ldots ,\tau_{q}\geq 0$ such that $\xi^{⊤}\left(F_{0}+F_{w}+F_{c}\right)\xi <0$ for all $\xi :={\left[x^{⊤}\;f^{⊤}\;w^{⊤}\right]}^{⊤}$, then $\xi^{⊤}\left(F_{0}+F_{w}\right)\xi <0$ for all ξ for which $-f_{i}\left(y_{i}\right)\left(f_{i}\left(y_{i}\right)-\sigma_{i}y_{i}\right)\geq 0,i=1,\ldots ,q$, namely for all $y_{i}$ satisfying the sector-bounded constraints. Hence, under $F_{0}+F_{w}+F_{c}<0$, condition (4) follows as claimed. □

## 4. State Feedback Optimal *L*_2_-Induced Control

Based on the lemma above one can derive the following result:

**Theorem** **1.** *If the following matrix inequality*

(9) $$\begin{array}{c} \left[\begin{array}{ccccc} M_{11}\left(X,Y\right) & M_{12}\left(X,Y,\Lambda ,T\right) & B_{1} & Y^{⊤}N^{⊤} & XM^{⊤}\\ {\left(1,\;2\right)}^{⊤} & \Lambda CF+F^{⊤}C^{⊤}\Lambda -T & \Lambda CB_{1} & 0 & 0\\ {\left(1,\;3\right)}^{⊤} & {\left(2,\;3\right)}^{⊤} & -\gamma^{2}I_{m_{1}} & 0 & 0\\ {\left(1,\;4\right)}^{⊤} & 0 & 0 & -I_{p} & 0\\ {\left(1,\;5\right)}^{⊤} & 0 & 0 & 0 & -I_{p} \end{array}\right]<0 \end{array}$$

*where*

$$\begin{array}{c} \begin{array}{ccc} M_{11}\left(X,Y\right) & := & XA^{⊤}+AX+Y^{⊤}B_{2}^{⊤}+B_{2}Y\\ M_{12}\left(X,Y,\Lambda ,T\right) & := & XA^{⊤}C^{⊤}\Lambda +Y^{⊤}B_{2}^{⊤}C^{⊤}\Lambda +F+\frac{1}{2}XC^{⊤}ST, \end{array} \end{array}$$

*is feasible with respect to $Y\in R^{m_{2}\times n}$, X>0, P>0, $\Lambda =\mathrm{diag}\{\lambda_{1},\ldots ,\lambda_{q}\}>0$ and $T=\mathrm{diag}\{\tau_{1},\ldots ,\tau_{q}\}>0$, then the state feedback control law u(t)=Kx(t) with $K=YX^{-1}$ stabilizes the system (1) and ensures the $L_{2}$ condition $\int_{0}^{\infty }\left(z^{⊤}\left(t\right)z\left(t\right)-\gamma^{2}w^{⊤}\left(t\right)w\left(t\right)\right)dt<0$ all $w\left(t\right)\in L^{2}\left(\left[0,\infty \right),R^{m_{1}}\right)$.*

**Proof.**  The proof directly follows applying Lemma 1 for $D=B_{1}$ and for $L=M+NB_{2}K$, multiplying the inequality (4) to the left and to the right by $\mathrm{diag}(P^{-1},I,I,I)$, denoting $X:=P^{-1}$ and $Y:=KP^{-1}$ and using Schur complement arguments. □

## 5. Loop Transformation and Reduced Conservatism

One can apply the result above for the synthesis of controllers for general nonlinear plants that can be represented by the system (1). We note that far more general models of plants can be represented in this manner, also in cases where system nonlinearities are not a priori sector-bounded. In such cases, one may invoke the universal approximation theorem [10] to systems where a single hidden layer, with, e.g., a *tanh* activation function and a linear output layer, provides an approximation with arbitrarily small error for an arbitrarily wide hidden layer. In such cases, the model of system (1) readily becomes relevant, as the approximate function is now sector-bounded. However, the formulation of (1) embeds possible conservatism, as one can express Ax+Ff=(A+FμC)x+F(f−μCx). In such a case, $\sigma_{i}$ are replaced by $\sigma_{i}-\mu$ provided $0<\mu <\sigma_{i}$.

We now aim at conservatism reduction. To this end, we first consider a non-symmetric sector bound. Recall that Ax+Ff=(A+μFC)x+F(f−μCx) and note that $\bar{f}=f-\mu Cx$ has shifted sector bounds. Since $f_{i}={\bar{f}}_{i}+\mu y_{i}$, the bounds $0\leq y_{i}f_{i}\leq \sigma_{i}y_{i}^{2}$ are replaced by

$$0\leq y_{i}\left({\bar{f}}_{i}+\mu y_{i}\right)\leq \sigma_{i}y_{i}^{2}$$

and get the following non-symmetric bounds as expected:

$$-\mu y_{i}^{2}\leq {\bar{f}}_{i}y_{i}\leq \left(\sigma_{i}-\mu \right)y_{i}^{2}.$$

Defining $\alpha_{i}=-\mu$ and $\beta_{i}=\sigma_{i}-\mu$, we finally obtain

(10) $$\begin{array}{c} \alpha_{i}y_{i}^{2}\leq y_{i}{\bar{f}}_{i}\leq \beta_{i}y_{i}^{2}. \end{array}$$

Multiplying by $y_{i}$ the inequalities (10), it results that ${\bar{f}}_{i}-\alpha_{i}y_{i}$ and $\beta_{i}y_{i}-\bar{f}i$ have the same sign, from which it follows that

(11) $$\begin{array}{c} y_{i}^{2}\left(\beta_{i}y_{i}-{\bar{f}}_{i}\right)\left({\bar{f}}_{i}-\alpha_{i}y_{i}\right)\geq 0. \end{array}$$

Conversely, if the above inequality holds, it follows that $\left(\beta_{i}y_{i}-{\bar{f}}_{i}\right)\left({\bar{f}}_{i}-\alpha_{i}y_{i}\right)\geq 0$, namely ${\bar{f}}_{i}-\alpha_{i}y_{i}$ and $\beta_{i}y_{i}-{\bar{f}}_{i}$ have the same sign, and therefore $y_{i}\left({\bar{f}}_{i}-\alpha_{i}y_{i}\right)$ and $y_{i}\left(\beta_{i}y_{i}-{\bar{f}}_{i}\right)$ have the same sign, too. If they are greater or equal than 0, the inequalities (10) directly follow. The case where both $y_{i}\left({\bar{f}}_{i}-\alpha_{i}y_{i}\right)$ and $y_{i}\left(\beta_{i}y_{i}-{\bar{f}}_{i}\right)$ are negative is easily shown to contradict the facts that $\alpha_{i}<0$ and $\beta_{i}>0$, i=1,…,q. Therefore, one concludes that (10) is equivalent to the condition $\left(\beta_{i}y_{i}-{\bar{f}}_{i}\right)\left({\bar{f}}_{i}-\alpha_{i}y_{i}\right)\geq 0$ for all i=1,…,q. Based on the definitions of $\alpha_{i}$ and $\beta_{i}$, i=1,…,q, one can define the matrices

$$\begin{array}{c} \alpha :=\mathrm{diag}(\alpha_{1},\ldots ,\alpha_{q})=-\mu I \end{array}$$

and

$$\begin{array}{c} \beta :=\mathrm{diag}(\beta_{1},\ldots ,\beta_{q})=S-\mu I. \end{array}$$

Taking into account that $y_{i}=C_{i}x,i=1,\ldots ,q$ where $C_{i}$ are the rows of *C*, direct algebraic computations show that the sum $\Sigma_{i=1}^{q}\tau_{i}\left(\beta_{i}y_{i}-{\bar{f}}_{i}\right)\left({\bar{f}}_{i}-\alpha_{i}y_{i}\right)$ required when applying the S-procedure is used can be expressed as ${\bar{\xi }}^{⊤}F_{c}^{\mu }\bar{\xi }$, where $\bar{\xi }:={\left[x^{T}\;{\bar{f}}^{T}\;w^{T}\right]}^{T}$ and where

(12) $$F_{c}^{\mu }:=\left[\begin{array}{ccc} \mu C^{⊤}\left(S-\mu I\right)TC & \frac{1}{2}C^{⊤}\left(S-2\mu I\right)T & 0\\ \frac{1}{2}T\left(S-2\mu I\right)C & -T & 0\\ 0 & 0 & 0 \end{array}\right].$$

We aim now at obtaining a version of Theorem 1 with the shifted sector bounds. To this end, we consider dV/dt of (6) and rewrite it as

$$\begin{array}{c} \left[\begin{array}{ccc} x^{⊤} & {\bar{f}}^{⊤}+\mu \;x^{⊤}C^{⊤} & w^{⊤} \end{array}\right]\;{\tilde{F}}_{0}\;\left[\begin{array}{c} x\\ \bar{f}+\mu \;Cx\\ w \end{array}\right]=\left[\begin{array}{ccc} x^{⊤} & {\bar{f}}^{⊤} & w^{⊤} \end{array}\right]\left(T_{\mu }^{⊤}{\tilde{F}}_{0}\;T_{\mu }\right)\left[\begin{array}{c} x\\ \bar{f}\\ w \end{array}\right], \end{array}$$

where

$$\begin{array}{c} T_{\mu }:=\left[\begin{array}{ccc} I & 0 & 0\\ \mu C & I & 0\\ 0 & 0 & I \end{array}\right], \end{array}$$

and where ${\tilde{F}}_{0}$ is obtained from (9) written for the system (1) with the control u=Kx, namely by replacing the matrices *A*, *L*, and *D* by $A+B_{2}K$, M+NK, and $B_{1}$, respectively. Using again the S procedure to impose the shifted sector bound constraints associated with ${\tilde{F}}_{0}^{\mu }:=T_{\mu }^{⊤}{\tilde{F}}_{0}T_{\mu }$, we readily obtain the loop-transformed version of condition (9) of Theorem 1,

(13) $$\begin{array}{c} {\tilde{L}}_{\mu }<0 \end{array}$$

where

(14) $$\begin{array}{c} {\tilde{L}}_{\mu }:=T_{\mu }^{⊤}{\tilde{F}}_{0}^{\mu }\;T_{\mu }+F_{c}^{\mu }+F_{w}. \end{array}$$

The latter inequality can be readily expressed using YALMIP [18], and we intentionally avoid writing the explicit formula for its blocks to avoid burdening the reader with unnecessary details. Note that similarly to the Lemma of Section 1, the search variables are P>0, $\Lambda =\mathrm{diag}(\lambda_{1},\ldots ,\lambda_{q})>0$ and $T=\mathrm{diag}(\tau_{1},\ldots ,\tau_{q})\geq 0$, where μ is found using line search within the interval $\mu \in [0,\min_{i}\sigma_{i}]$.

Based on Theorem 1, one obtains the following result.

**Theorem** **2.** *If there exist matrices P>0, diagonal matrices Λ,T>0, a matrix K and a scalar $\mu \in [0,\min_{i}\sigma_{i}]$ such that*

(15) $$\begin{array}{c} \left[\begin{array}{ccc} N_{11}\left(P,\Lambda ,K,\mu \right) & N_{12}\left(P,\Lambda ,T,K,\mu \right) & N_{13}\left(P,\Lambda ,\mu \right)\\ {\left(1,\;2\right)}^{⊤} & N_{22}\left(\Lambda ,T\right) & N_{23}\left(\Lambda \right)\\ {\left(1,\;3\right)}^{⊤} & {\left(2,\;3\right)}^{⊤} & -\gamma^{2}I_{m_{1}} \end{array}\right]<0 \end{array}$$

*in which*

$$\begin{array}{c} \begin{array}{ccc} N_{11}\left(P,\Lambda ,K,\mu \right) & = & {\left(A+B_{2}K\right)}^{⊤}P+P\left(A+B_{2}K\right)+{\left(M+NK\right)}^{⊤}\left(M+NK\right)\\ & & +\mu C^{⊤}\left[\Lambda C\left(A+B_{2}K\right)+F^{⊤}P\right]+\mu \left[{\left(A+B_{2}K\right)}^{⊤}C^{⊤}\Lambda +PF\right]C\\ & & +\mu^{2}C^{⊤}\left(\Lambda CF+F^{⊤}C^{⊤}\Lambda \right)C+\mu C^{⊤}\left(S-\mu I\right)TC\\ N_{12}\left(P,\Lambda ,T,K,\mu \right) & = & {\left(A+B_{2}K\right)}^{⊤}C^{⊤}\Lambda +PF+\mu C^{⊤}\left(\Lambda CF+F^{⊤}C^{⊤}\Lambda \right)\\ & & +\frac{1}{2}C^{⊤}\left(S-2\mu I\right)T\\ N_{13}\left(P,\Lambda ,\mu \right) & = & \left(P+\mu C^{⊤}\Lambda C\right)B_{1}\\ N_{22}\left(\Lambda ,T\right) & = & \Lambda CF+F^{⊤}C^{⊤}\Lambda -T\\ N_{22}\left(\Lambda \right) & = & \Lambda CB_{1}, \end{array} \end{array}$$

*then K stabilizes the system (1) satisfying the γ-attenuation condition $\int_{0}^{\infty }\left(z^{T}\left(t\right)z\left(t\right)-\gamma^{2}w^{T}\left(t\right)w\left(t\right)\right)$dt<0 for all $w\left(t\right)\in L^{2}\left(\left[0,\infty \right),R^{m_{1}}\right)$.*

The inequality (15) above is nonlinear. Further representation of (15) for numerical implementation now follows. Fixing Λ and *T* and multiplying the inequality (15) to the left and to the right by $\mathrm{diag}\{P^{-1},I,I\}$, and denoting $X:=P^{-1}$ and $Y:=KP^{-1}$, based on Schur complement arguments and using the inequality $XC^{⊤}\Lambda CB_{2}Y+Y^{⊤}B_{2}^{⊤}C^{⊤}\Lambda CX\leq XC^{⊤}\Lambda CX+Y^{⊤}B_{2}^{⊤}C^{⊤}\Lambda CB_{2}Y$, it results that the condition (15) is accomplished if the following linear matrix inequality (LMI) is feasible with respect to X>0 and *Y*:

(16) $$\begin{array}{c} \left[\begin{array}{ccccc} P_{11}\left(X,Y\right) & P_{12}\left(X,Y\right) & P_{13}\left(X\right) & P_{14}\left(X\right) & P_{15}\left(Y\right)\\ {\left(1,\;2\right)}^{⊤} & P_{22} & P_{23} & 0 & 0\\ {\left(1,\;3\right)}^{⊤} & {\left(2,\;3\right)}^{⊤} & -\gamma^{2}I_{m_{1}} & 0 & 0\\ {\left(1,\;4\right)}^{⊤} & 0 & 0 & -I_{n} & 0\\ {\left(1,\;5\right)}^{⊤} & 0 & 0 & 0 & -I_{m_{2}} \end{array}\right]<0, \end{array}$$

where

(17) $$\begin{array}{c} \begin{array}{ccc} P_{11}\left(X,Y\right) & = & \left(A+\mu FC\right)X+X{\left(A+\mu FC\right)}^{⊤}+B_{2}Y+Y^{⊤}B_{2}^{⊤}\\ P_{12}\left(X,Y\right) & = & X\left[A^{⊤}C^{⊤}\Lambda +\mu C^{⊤}\left(\Lambda CF+F^{⊤}C^{⊤}\Lambda \right)\right]+Y^{⊤}B_{2}^{⊤}C^{⊤}\Lambda +F\\ & & +\frac{1}{2}XC^{⊤}\left(S-2\mu I\right)T\\ P_{13}\left(X\right) & = & \left(I+\mu XC^{T}\Lambda C\right)B_{1}\\ P_{14}\left(X\right) & = & XV^{⊤}\\ P_{15}\left(Y\right) & = & Y^{⊤}W^{⊤}\\ P_{22} & = & \Lambda CF+F^{⊤}C^{⊤}\Lambda -T\\ P_{23} & = & \Lambda CB_{1} \end{array} \end{array}$$

and where the matrices *V* and *W* satisfy the following conditions:

(18) $$\begin{array}{c} \begin{array}{ccc} V^{⊤}V & = & M^{⊤}M+\mu \left[C^{⊤}\Lambda CA+A^{⊤}C^{⊤}\Lambda C+C^{⊤}\left(S-\mu I\right)TC+C^{⊤}\Lambda C\right]\\ & & +\mu^{2}\left(C^{⊤}\Lambda CF+F^{⊤}C^{⊤}\Lambda C\right)\\ W^{⊤}W & = & N^{⊤}N+\mu B_{2}^{⊤}C^{⊤}\Lambda CB_{2}. \end{array} \end{array}$$

**Remark** **1.** *Note that Theorem 2 involves terms that are bilinear in* Λ *and T that were assumed to be fixed, or, in other words, remained for manual choice. One can, however, embed (16) in an optimization scheme, where the diagonal terms in* Λ *and T are vectorized. One can then minimize a smooth approximation of max(0, $\lambda_{max}\left(P\right)$). While such an approach is free from manual tuning of* Λ *and T, one should keep in mind that global convergence of such a heuristic approach does not guarantee global feasibility. Nevertheless, the results of Section 6 below employ such an approach.*

## 6. State Feedback Control with *L*_2_-Induced Norm Attenuation for Van Der Pol Oscillator

The theoretical results derived in the previous sections are illustrated for a van der Pol oscillator described by the differential equation:

(19) $$\begin{array}{c} \ddot{x}\left(t\right)-\epsilon \left(1-x^{2}\left(t\right)\right)\dot{x}\left(t\right)+x\left(t\right)=0 \end{array}$$

where ϵ≥0 is a fixed parameter indicating the strength of the nonlinear damping. As it is well-known, this (unforced) equation was introduced a century ago in the context of oscillations modeling in a vacuum tube electrical circuit. Since then, it has been shown that such an equation is useful not only in electronics but also in other diverse domains as physics, biology, neurology and more recently in machine learning and evolutionary algorithms used to represent the real electrocardiographic signals ([19]). The differential Equation (19) may be represented in the state space by letting $x_{1}:=x$ and $x_{2}:=\dot{x}$ for which one gets

(20) $$\begin{array}{c} \begin{array}{ccc} {\dot{x}}_{1}\left(t\right) & = & x_{2}\left(t\right)\\ {\dot{x}}_{2}\left(t\right) & = & \epsilon \left(1-x_{1}^{2}\left(t\right)\right)x_{2}\left(t\right)-x_{1}\left(t\right). \end{array} \end{array}$$

Since the aim of this application is to stabilize (20), ensuring a certain prescribed level of attenuation of the $L_{2}$-induced norm, one considered the following modified (forced) form of (20):

(21) $$\begin{array}{c} \begin{array}{ccc} {\dot{x}}_{1}\left(t\right) & = & x_{2}\left(t\right)+w\left(t\right)\\ {\dot{x}}_{2}\left(t\right) & = & \epsilon \left(1-x_{1}^{2}\left(t\right)\right)x_{2}\left(t\right)-x_{1}\left(t\right)+u\left(t\right), \end{array} \end{array}$$

where $w\left(t\right)\in L^{2}\left(\left[0,\infty \right),R\right)$ is a disturbance input and u(t)∈R denotes the control input.

**Figure 1:**
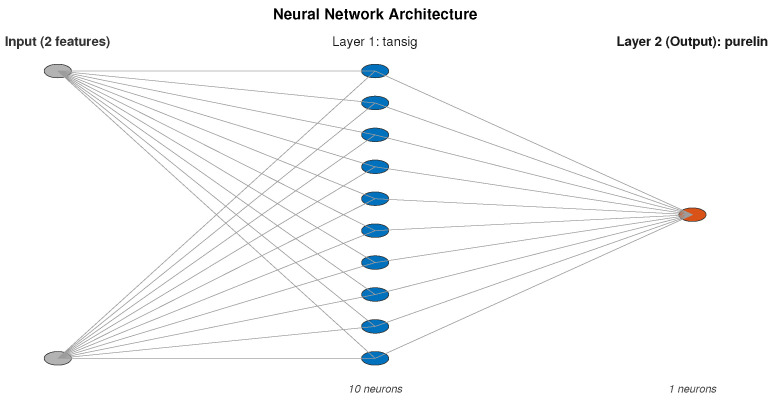
Neural Network.

As can be seen, the nonlinearity in (20) is not a sector-type one; it, therefore, was approximated using the universal approximation theorem proved by Cybenko [10], stating that any continuous function can be approximated arbitrarily well by a neural network with at least one hidden layer with a finite number of weights. In the present application, the nonlinear term $\epsilon \left(1-x_{1}^{2}\right)x_{2}$ was approximated as

(22) $$\begin{array}{c} \epsilon \left(1-x_{1}^{2}\right)x_{2}\approx \sum_{i=1}^{N}\left[W_{2,i}\left(\tanh\left(W_{1,i}\left[\begin{array}{c} x_{1}\\ x_{2} \end{array}\right]+b_{1,i}\right)\right)+b_{2,i}\right], \end{array}$$

with *N* denoting the number of neurons and where the weights and the bias terms were determined by back propagation training (see e.g., [8]). The network architecture is depicted in Figure 1.

**Figure 2:**
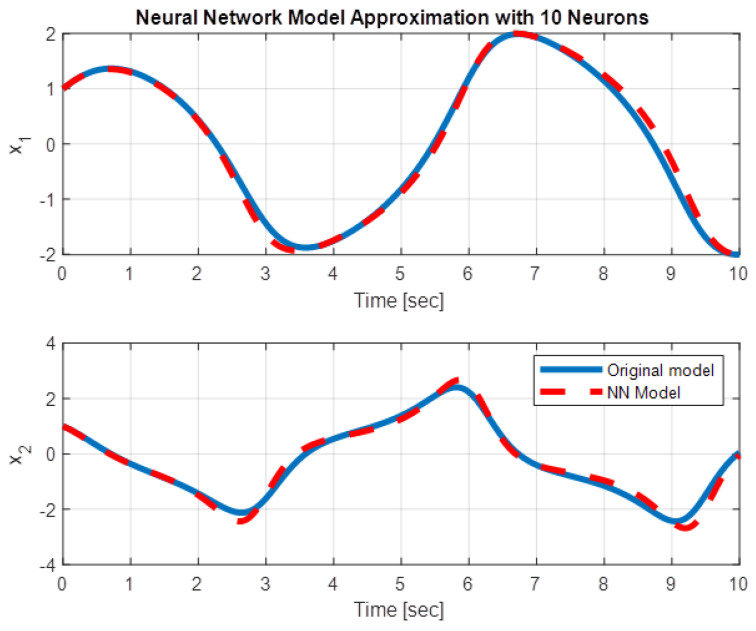
Time responses of the states: blue—the states of the unforced oscillator (20); red—the states of the oscillator with the approximation (22).

**Figure 3:**
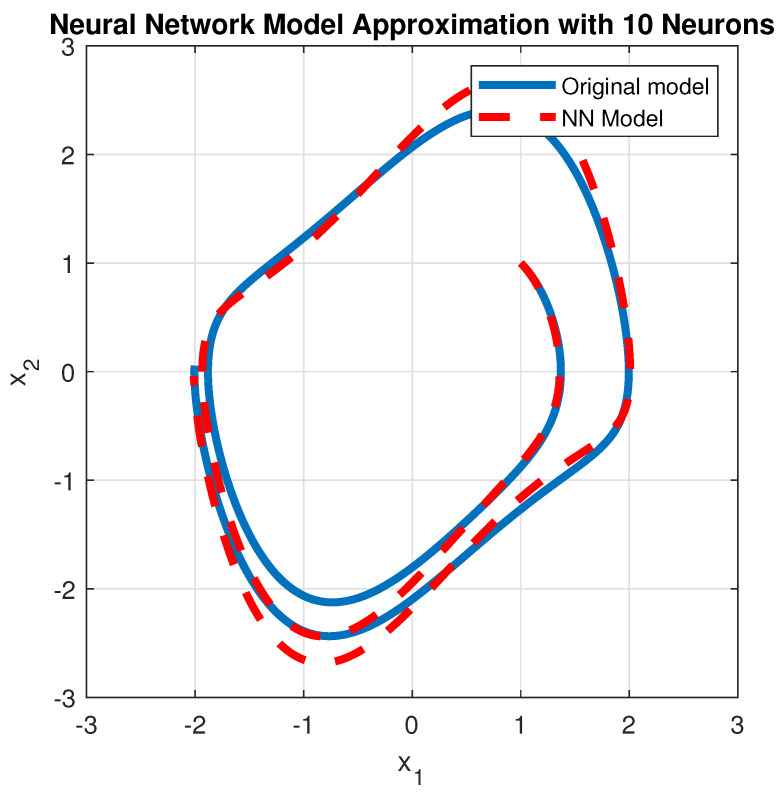
Phase–Plane responses: blue—the states of the unforced oscillator (20); red—the states of the oscillator with the approximation (22).

For ϵ=1 and for N=10, the time responses obtained with the approximation (22) of $\epsilon \left(1-x_{1}^{2}\right)x_{2}$ are presented in Figure 2 (states as a function of time) and Figure 3 (phase–plane), comparatively with the time responses of the original van der Pol oscillator (20).

Using the approximation above of the nonlinearity $\epsilon \left(1-x_{1}^{2}\right)x_{2}$ and defining

(23) $$\begin{array}{c} A=\left[\begin{array}{cc} 0 & 1\\ -1 & 0 \end{array}\right],\;\;B_{1}=\left[\begin{array}{c} 1\\ 0 \end{array}\right],\;B_{2}=\left[\begin{array}{c} 0\\ 1 \end{array}\right],\;\;F=\left[\begin{array}{c} 0_{1\times N}\\ W_{2,1}\ldots W_{2,N} \end{array}\right], \end{array}$$

it follows that the van der Pol oscillator (21) may be approximated as the first Equation (1), where $f_{i}\left(y_{i}\right)=tanh\left(W_{1,i}\left[\begin{array}{c} x_{1}\\ x_{2} \end{array}\right]\right)$ and therefore, $y_{i}=W_{1,i}\left[\begin{array}{c} x_{1}\\ x_{2} \end{array}\right]$, i=1,…,N. Following the notations of (2) it results that $C_{i}=W_{1,i},i=1,\ldots ,N$.

As far as the quality output is concerned, one considers $z={\left[\begin{array}{cc} x_{1} & 0.1u \end{array}\right]}^{⊤}$, obtaining thus the matrices *M* and *N* from the second Equation (1) as

(24) $$\begin{array}{c} M=\left[\begin{array}{cc} 1 & 0\\ 0 & 0 \end{array}\right],\;\;N=\left[\begin{array}{c} 0\\ 0.1 \end{array}\right]. \end{array}$$

**Figure 4:**
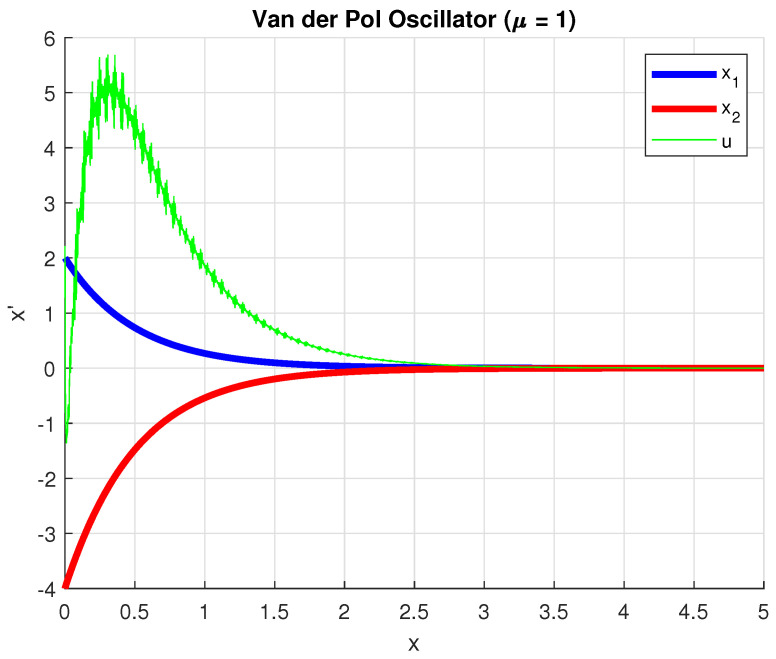
Time responses of the states of the forced oscillator (20); blue—$x_{1}$, red—$x_{2}$, green—*u*.

Note that (16) is convex only for fixed Λ and *T* for a given μ. Therefore, we resorted to using unconstrained minimization of a scalar objective using the derivative-free Nelder–Mead simplex method (i.e., fminsearch from MATLAB version 25.1 (R2025a) ) starting from an initial guess provided by solving the LMI of (16) for a guess of Λ, *T* and μ and for γ=500 for which the LMI was solved. The search variables in this optimization scheme are the vectorized versions vec(Y) and vech(X), respectively, of *X* and *Y*. Then, $K=YX^{-1}$ it results that K=[−662.3694 −331.7394]. The closed-loop simulation results with this gain are plotted in Figure 4.

## 7. Conclusions

Boundedness conditions for the $L_{2}$-induced norm of systems with multiple bounded-sector nonlinearities have been derived in the present paper. Based on these conditions, a state feedback optimal control problem was formulated and solved. The solvability conditions were expressed in terms of the feasibility of a specific system of matrix inequalities. A loop transformation procedure was developed in order to reduce the conservatism of the sufficient solvability conditions. The problem considered in the present paper does not represent just an extension of optimal control for Lur’e systems to the case with multiple bounded-sector nonlinearities. It was shown that in combination with the universal approximation theorem, these results may be successfully used for optimal control problems with nonlinearities that do not necessarily satisfy sector-bounded constraints. This idea was illustrated for the optimal $L_{2}$ control problem of a forced van der Pol oscillator, but the proposed design procedure may be used for many other types of nonlinearities arising in applications. Further applications of Lur’e systems and the strongly related continuous-time Hopfield networks may utilize alternative, other than quadratic, energy functions (see [20]) and are left for future research.

## Data Availability

The original contributions presented in this study are included in the article. Further inquiries can be directed to the corresponding author.
